# Decision making processes based on social conventional rules in early adolescents with and without autism spectrum disorders

**DOI:** 10.1038/srep37875

**Published:** 2016-11-29

**Authors:** Hidetsugu Komeda, Hidekazu Osanai, Kaichi Yanaoka, Yuko Okamoto, Toru Fujioka, Sumiyoshi Arai, Keisuke Inohara, Masuo Koyasu, Takashi Kusumi, Shinichiro Takiguchi, Masao Kawatani, Hirokazu Kumazaki, Michio Hiratani, Akemi Tomoda, Hirotaka Kosaka

**Affiliations:** 1 The Hakubi Center for Advanced Research, Kyoto University, Kyoto 606-8501, Japan; 2 Division of Cognitive Psychology in Education, Graduate School of Education, Kyoto University, Kyoto 606-8501, Japan; 3 Research Center for Child Mental Development, University of Fukui, Eiheiji-cho, Fukui 910-1193, Japan; 4 Department of Child and Adolescent Psychological Medicine, University of Fukui Hospital, 23-3 Matsuoka-Shimoaizuki, Eiheiji-cho, Fukui 910-1193, Japan; 5 Department of Neuropsychiatry, Faculty of Medical Sciences, University of Fukui, Eiheiji-cho, Fukui 910-1193, Japan; 6 Department of Informatics, Graduate School of Informatics and Engineering, The University of Electro-Communications, Chofu, Tokyo 182-8585, Japan; 7 Japan Society for the Promotion of Science, Kojimachi Business Center Building, 5-3-1 Kojimachi, Chiyoda-ku, Tokyo 102-0083, Japan; 8 Department of Human Sciences, Faculty of Letters, Konan University, Kobe, 658-8501, Japan; 9 Department of Pediatrics, Faculty of Medical Sciences, University of Fukui, Eiheiji-cho, Fukui, 910-1193, Japan; 10 Hiratani Clinic for Developmental Disorders of Children, Fukui, 918-8205, Japan

## Abstract

Autism spectrum disorder (ASD) is characterized by problems with reciprocal social interaction, repetitive behaviours/narrow interests, and impairments in the social cognition and emotional processing necessary for intention-based moral judgements. The aim of this study was to examine the information used by early adolescents with and without ASD when they judge story protagonists as good or bad. We predicted that adolescents with ASD would use protagonists’ behaviour, while typically developing (TD) adolescents would use protagonists’ characteristics when making the judgements. In Experiment 1, we measured sentence by sentence reading times and percentages for good or bad judgements. In Experiment 2, two story protagonists were presented and the participants determined which protagonist was better or worse. Experiment 1 results showed that the adolescents with ASD used protagonist behaviours and outcomes, whereas the TD adolescents used protagonist characteristics, behaviours, and outcomes. In Experiment 2, TD adolescents used characteristics information when making “bad” judgements. Taken together, in situations in which participants cannot go back and assess (Experiment 1), and in comparable situations in which all information is available (Experiment 2), adolescents with ASD do not rely on information about individual characteristics when making moral judgements.

Moral psychology research has focused on two perspectives or models: Rationalist and intuitionist. The rationalist claims that moral knowledge and moral judgements are the product of a conscious process of reasoning and reflection^1^,^2^,^3^. The intuitionist claims that moral intuitions, such as unconscious moral emotions, occur immediately in situations and directly cause moral judgements^4^,^5^,^6^,^7^. In addition, knowing the facts of a situation changes the emotional response and the moral judgement that one makes^8^. For example, anger that a student failed to show up for an exam can turn to sympathy when one discovers that the reason for the absence was a death in the family^9^. Thus, situational as well as conscious and unconscious individual factors should be considered in investigations of moral judgements.

Autism spectrum disorder (ASD) is characterized by problems with reciprocal social interaction, impaired communication, repetitive behaviours/narrow interests^10^, and impairments in the social cognition and emotional processing necessary for intention-based moral judgements^11^,^12^. It is widely reported that individuals with ASD who have comorbid alexithymia are atypical in terms of recognizing their own emotions^13^,^14^ and in empathizing with others^15^,^16^,^17^. Empathy is a social emotion that plays a crucial role in such moral evaluations, because empathy allows moral judges to understand suffering and to use the resulting “gut-feelings” to either approve or disapprove of moral actions^18^,^19^.

According to social domain theory^3^, children develop not only moral concepts but also concepts of social convention. Moral concepts are related humans’ natural aversion to harming others and natural attraction to helping others^20^,^21^, whereas social convention would involve no direct harm or victimization^3^. The distinction between moral and conventional domains is still debated^22^. Although recent studies have shown that moral cognition is based on conscious and unconscious processes^22^, the corresponding conscious and unconscious processes involved in social convention remain unclear. Decision-making processes based on social conventional rules have been shown to involve one or more of three possible components: Trait inferences based on characteristics^23^, inferences based on behaviours^24^, and evaluation of outcomes as outputs^25^. Typically developing individuals in early adolescence make trait inferences as well as behaviour- and intention-based inferences^26^. Individuals with ASD evidence deficits in more complex intention-based moral judgement tasks that require integration of information about agent mental states with information about the outcomes of behaviours^19^. Recent studies using IAT (implicit association test) tasks have shown that implicit social biases are largely intact in ASD^23^, suggesting that individuals with ASD do have the ability to make trait inferences. Children with ASD can also make behaviour-based inferences. They are able to make correct moral judgements about so-called naughty behaviour, although their judgement criteria are more rigid^24^. Similarly, children with ASD can have knowledge of social rules but are less flexible than typically developing (TD) children in applying these rules during moral reasoning^25^. Adolescents with ASD are also able to evaluate outcomes of moral decision-making processes^27^. Although adults with ASD have difficulty assessing the seriousness of moral and disgust-based transgressions and providing appropriate welfare-based moral justifications^28^, they do not appear to exhibit fundamental deficits in moral judgements^29^. At least under certain circumstances, individuals with ASD and typically developing individuals can provide similar intuitive judgements of intentionality^30^.

We tend to make up reasons post-hoc for the conflicting intuitions that arise within us^31^,^32^. Children with autism are known to have difficulty in solving social problems, particularly in conflict resolution^33^. This suggests that they may have difficulty in cases of conflict between traits and behaviours, rather than problems in separately making trait inferences, behavioural inferences, and outcome evaluations. The present study examines the effect of (in) congruency between traits and behaviours on moral judgements, as well as the separate effects of trait and behavioural inferences, by creating discrepancies in the information about story characters that resemble social conflict situations.

Previous studies have typically used short stories involving situations in which the characters are at risk of harm. Moral dilemmas are often presented as variations of the basic trolley, bystander, or footbridge scenarios^34^, and two situations are presented to clarify the nature of the transgression believed to make the situation worse. In the final part of the story, an alternative action is proposed in the form of a commission or omission^35^. This alternative course typically results in less harm than the original bad outcome in terms of overall quantitative outcome, or in terms of providing other benefits^2^. Such studies have provided valuable information about moral judgements in the case of negative outcomes. However, in daily life, positive or fun events occur as well as negative or harmful ones. Yet there have been few studies about moral judgements in the case of good outcomes^36^,^37^. The present study investigates decision-making processes based on social conventional rules, assessing both positive and negative aspects of a protagonist’s characteristics, behaviours, and story outcomes.

The fundamental aim of this study was to examine the information early adolescents use when they judge other children’s behaviour as good or bad based on social conventional norms. We predicted that adolescents with ASD would rely on protagonists’ behaviour, whereas TD adolescents would rely more on protagonist characteristics during the decision-making processes. To identify the types of information upon which adolescents with and without ASD rely, we created simple, novel stories in which the protagonist’s characteristics and behaviours, as well as story outcomes, were manipulated. In Experiment 1, we measured sentence by sentence reading times and good or bad judgements. Our immediate moral intuitions are made via conscious deliberation, and such deliberation plays a central role in our moral judgements^8^. Thus, in Experiment 2, two protagonists were presented in conscious and comparable situations to determine which protagonist is better or worse.

## Experiment 1

### Method

#### Participants

##### Sample-size determination

Twenty adolescents with ASD and twenty TD adolescents were recruited. We followed recommended guidelines^38^ and calculated our target sample size using an estimated effect size, *d*, of 0.45^39^, which would require a sample size of approximately 40 participants for the study to have 80% power. Because the data for one adolescent with ASD were lost, we analyzed nineteen adolescents with ASD (mean age = 12.5 years) and twenty age- and IQ- matched TD adolescents (mean age = 12.3 years; see Table 1).

Adolescents with ASD were recruited at the Department of Neuropsychiatry of the University of Fukui Hospital, Japan, and the Department of Psychiatry and Hiratani Clinic for Developmental Disorders of Children, Japan. Pediatric psychiatrists (M.H. and H.K.) diagnosed participants based on the Diagnostic and Statistical Manual of Mental Disorders (DSM-5^10^) and on the standardized criteria of the Diagnostic Interview for Social and Communication Disorders^40^, which is reported to possess good psychometric properties^41^. This instrument also contains items on early development and a section on activities of daily living, which provide data about functioning in areas other than social- and communication-related domains^40^. The ASD group consisted of 19 participants (2 females and 17 males).

TD adolescents were recruited in the local community and were matched to the adolescents with ASD based on age and gender. A total of 39 participants completed the Wechsler Intelligence Scale for Children—Fourth Edition (WISC-IV)^42^, for assessment of intelligence quotient (IQ). The TD group consisted of 20 participants (2 females and 18 males).

All participants were financially compensated for their time and informed consent from all participants and their parents was obtained. All methods were carried out in accordance with the approved guidelines. The study was approved by the local Ethics Committees (Kyoto University and the University of Fukui) and conducted in accordance with the declaration of Helsinki.

##### Stimuli and procedure

As shown in Table 2, each story consisted of three sentences (first sentence: characteristics; second sentence: behaviours; and third sentence: outcomes). The number of letters in the third sentences was identical across all stories so that reading times were comparable across conditions. The stories were presented one sentence at a time on a computer screen. Each sentence remained on the screen until the participant pressed the space bar, and then the next sentence appeared. Thus, participants could not refer back to the previous sentences but could read the sentences at their own pace. The computer recorded reading times for each sentence.

After reading each story, participants judged the story protagonists as good or bad. Participants read 24 stories, 3 for each combination of 2 characteristics (good, bad) × 2 behaviours (good, bad) × 2 outcomes (good, bad). Before the formal trials, the participants were given two practice stories to familiarize themselves with the reading procedure.

## Results

Analyses of covariance (ANCOVAs) were conducted to assess for interaction effects of working memory and language comprehension, controlling for IQ scores based on WISC-IV^42^ as a covariates of the dependent variables of reading times and judgements. Results indicated no significant interaction effects of working memory and language comprehension. Thus, we proceeded with our analysis without including covariates and report these results here.

### Reading time results for sentence by sentence task

**Figure 1:**
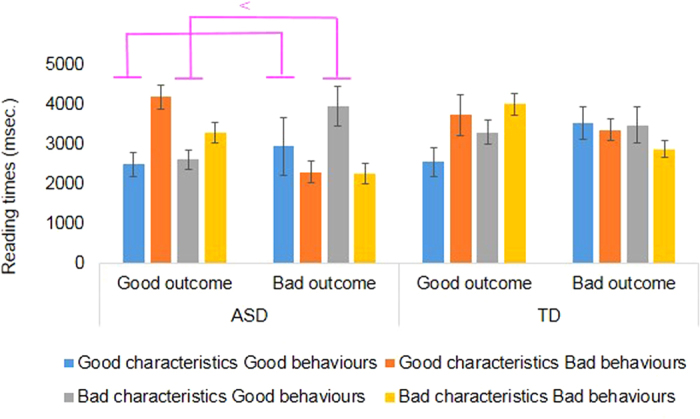
Reading times for target sentences in Experiment 1. The blue bars show good characteristics with good behaviours, the dark orange bars show good characteristics with bad behaviours, the gray bars show bad characteristics with good behaviours, and the light orange bars show bad characteristics with bad behaviours. Error bars represent 95% confidence intervals.

We conducted a group (ASD vs. TD) × characteristics × behaviours × outcomes analysis of variance (ANOVA) for reading times of the target sentences (Fig. 1). The group × characteristics × outcomes interaction was significant, *F* (1, 37) = 8.34, *p* = 0.01, *η*_*p*_^*2*^ = 18, Simple effects clarified the differences separately for each group (Table 3).

In the ASD group, the characteristics × behaviours interaction was significant, *F* (1, 18) = 5.87, *p* = 0.03, *η*_*p*_^*2*^ = 0.25. Post-hoc test results showed that the reading times for good characteristics with good behaviours (2725.0 msec.) were faster than those for bad characteristics with good behaviours (3295.3 msec.; *F* (1, 18) = 7.20, *p* = 0.02, *η*_*p*_^*2*^ = 0.29). The characteristics × outcomes interaction was also significant for the ASD group, *F* (1, 18) = 4.46, *p* = 0.05, *η*_*p*_^*2*^ = 0.20. Reading times for good characteristics with good outcomes (3348.9 msec.) were longer than those for good characteristics with bad outcomes (2628.2 msec.; *F* (1, 18) = 5.54, *p* = 0.03, *η*_*p*_^*2*^ = 0.24), and the reading times for bad outcomes with bad characteristics (3121.4 msec.) were longer than those for bad outcomes with good characteristics (2628.2 msec.; *F* (1, 18) = 4.65, *p* = 0.04, *η*_*p*_^*2*^ = 0.21). Finally, the behaviours × outcomes interaction was also significant for the ASD group, *F* (1, 18) = 21.48, *p* = 0.00, *η*_*p*_^*2*^ = 0.54. Reading times for good behaviours with good outcomes were faster than those for good behaviours with bad outcomes, *F* (1, 18) = 13.50, *p* = 0.00, *η*_*p*_^*2*^ = 0.43, the reading times for bad behaviours with bad outcomes were faster than those for bad behaviours with good outcomes, *F* (1, 18) = 16.04, *p* = 0.00, *η*_*p*_^*2*^ = 0.47, the reading times for good outcomes with good behaviours were faster than those for good outcomes with bad behaviours, *F* (1, 18) = 10.86, *p* = 0.00, *η*_*p*_^*2*^ = 0.38, and the reading times for bad outcomes with bad behaviours were faster than those for bad outcomes with good behaviours, *F* (1, 18) = 26.94, *p* = 0.00, *η*_*p*_^*2*^ = 0.60.

For the TD group (Table 3), the main effect of behaviours was significant, *F* (1, 19) = 6.45, *p* = 0.02, *η*_*p*_^*2*^ = 0.25, and the reading times for bad behaviours were longer than those for good behaviours. The behaviours × outcomes interaction was also significant, *F* (1, 19) = 15.19, *p* = 0.00, *η*_*p*_^*2*^ = 0.44. Post-hoc test results showed that reading times for good behaviours with good outcomes were faster than those for good behaviours with bad outcomes, *F* (1, 19) = 14.71, *p* = 0.00, *η*_*p*_^*2*^ = 0.44, the reading times of bad behaviours with bad outcomes were faster than those for bad behaviours with good outcomes, *F* (1, 19) = 5.40, *p* = 0.03, *η*_*p*_^*2*^ = 0.22, reading times for good outcomes with good behaviours were faster than those for good outcomes with bad behaviours, *F* (1, 19) = 14.02, *p* = 0.00, *η*_*p*_^*2*^  = 0.42, and reading times for bad outcomes with bad behaviours were faster than those for bad outcomes with good behaviours, *F* (1, 19) = 8.04, *p* = 0.01, *η*_*p*_^*2*^ = 0.30.

### Analyses of judgements

**Figure 2:**
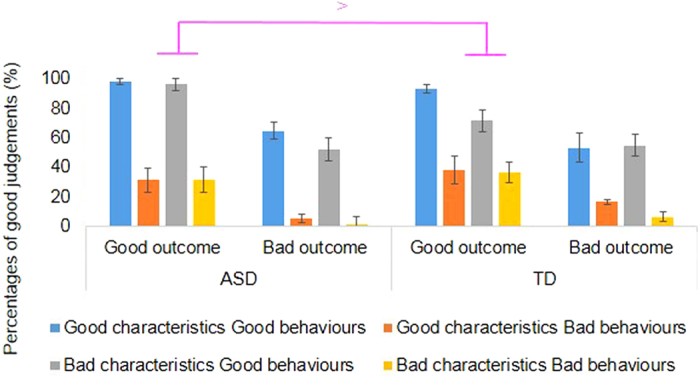
Percentages of “good” judgements in Experiment 1. The blue bars show good characteristics with good behaviours, the dark orange bars show good characteristics with bad behaviours, the gray bars show bad characteristics with good behaviours, and the light orange bars show bad characteristics with bad behaviours. Error bars represent 95% confidence intervals.

We next conducted a group × characteristics × behaviours × outcomes ANOVA based on percentages of good judgements (Fig. 2). The interaction between group × outcomes × characteristics × behaviour on good or bad judgements was significant, *F* (1, 37) = 4.33, *p* = 0.04, *η*_*p*_^*2*^ = 0.10. Simple effects revealed the differences in each group (see Table 4). In the ASD group, the main effects of behaviours (*F* (1, 18) = 129.16, *p* = 0.00, *η*_*p*_^*2*^ = 0.88) and outcomes (*F* (1, 18) = 38.53, *p* = 0.00, *η*_*p*_^*2*^ = 0.68) were significant. In the TD group (Table 4), the main effects of characteristics (*F* (1, 19) = 4.83, *p* = .04, *η*_*p*_^*2*^ = 0.20), behaviours (*F* (1, 19) = 80.72, *p* = 0.00, *η*_*p*_^*2*^ = 0.81) and outcomes were significant (*F* (1, 19) = 26.11, *p* = 0.00, *η*_*p*_^*2*^ = 0.58). The characteristics × behaviours × outcomes interaction was also significant, *F* (1, 19) = 6.73, *p* = 0.02, *η*_*p*_^*2*^ = 0.26. In the judgement of a person with “bad characteristics, good behaviours, with good outcomes”, the ASD group (96.5%) judged “Good person” more than the TD group did (71.7%); *F* (1, 37) = 10.90, *p* = 0.00, *η*_*p*_^*2*^ = 0.23.

## Discussion

The interaction between behaviours and outcomes on reading times showed that, for both the ASD and TD groups, the reading times for good behaviours with good outcomes were faster than those for good behaviours with bad outcomes, the reading times for bad behaviours with bad outcomes were faster than those for bad behaviours with good outcomes, the reading times for good outcomes with good behaviours were faster than those of good outcomes with bad behaviours, and that the reading times for bad outcomes with bad behaviours were faster than those for bad outcomes with good behaviours. Thus, groups showed congruency effects between behaviours and outcomes in the sentence-by-sentence reading task. The congruency effects are likely observed as a function of updating previous information^43^. If the behaviours and outcomes share the same valence, the processing load to update the information is relatively low, and thus reading times do not increase. However, if the behaviours and outcomes have opposite valences, the processing load to update the information is high, with a corresponding increase in reading times^44^.

The interaction between characteristics and behaviours was significant only for the ASD group. This suggests that the ASD group focused on the congruencies between characteristics and behaviours, particularly when reading about protagonists with bad characteristics and good behaviours. This effect did not reach a statistically significant level for protagonists with bad characteristic and good behaviours, when protagonists with bad characteristic and bad behaviours served as the comparison.

Although the interaction between characteristics and outcomes was significant for the ASD group, the congruency effects between characteristics and outcomes were not observed (i.e., reading times for good characteristics with good outcomes were not faster than those for good characteristic with bad outcomes, and reading times for bad outcomes with bad characteristics were not faster than those for bad outcomes with good characteristics). Thus, in both the ASD and TD groups, the congruencies between protagonist characteristics and story outcomes were not applied to the judgement processes.

The TD group appeared to focus on the behavioural information provided, given a significant main effect of behaviours. In the judgement processes, situational factors are important^8^,^9^. The TD group took situational factors behind the protagonist’s bad behaviours into consideration.

Overall, the ASD group used behaviours and outcome information to render their judgements, whereas the TD group used characteristics, behaviours, and outcome information in these decision-making processes based on social conventional rules. The ASD group did not appear to use the characteristics information. This finding is not explained by differences in working memory and language comprehension abilities, as ANVOCA indicated no significant interaction effects due to these covariates.

The ASD group was more likely to judge “good person” in the case of a protagonist with “bad characteristics, good behaviours, with good outcomes”, compared to the TD group. Thus, adolescents with ASD judged protagonists as good when their behaviour was good, whether or not the protagonists also had bad characteristics. However, this pattern was observed for the “good” outcomes only.

The adolescents with ASD focused on the congruencies between the characteristic and behaviours as well as the congruencies between the behaviours and the outcome. Thus, they were engaged in the updating process twice. However, the TD adolescents focused on the congruencies between the behaviours and the outcome. Thus, they engaged in only a single updating process. These findings highlight a strategic difference between ASD and TD adolescents with regards to moral decision-making. Although the adolescents with ASD studied here had the same working memory abilities as TD adolescents, these adolescents relied on a relatively inefficient judgement strategy. This updating load likely prevents adolescents with ASD from using the available characteristics information to render their judgements, while TD adolescents can use the characteristics information based on use of more efficient strategies involving a minimum updating load.

We predicted that adolescents with ASD use protagonists’ behaviour, while TD adolescents use protagonists’ characteristics in the judgements. This prediction was partially supported, such that the adolescents with ASD used behaviours and outcomes, while the TD adolescents used the characteristics, the behaviours, and outcomes.

## Experiment 2

In Experiment 1, a sentence-by-sentence reading paradigm was used, such that participants could not re-read previously presented sentences. Although this paradigm was useful in investigating integration processes during ongoing judgements, accessibility was not controlled: Behavioural information (the second sentence) was more proximate to the outcome than characteristics (first sentence) because of the fixed presentation order of the sentences.

In Experiment 2, all sentences were presented at the same time to control accessibility. This enables the participants to compare the different type of stories when both the characteristics and behaviours are available at the same time. In this way we can identify the information that is relied upon by the participants when making their moral decisions.

### Method

#### Participants

All the participants who completed Experiment 1 also completed Experiment 2.

##### Stimuli and procedure

**Figure 3:**
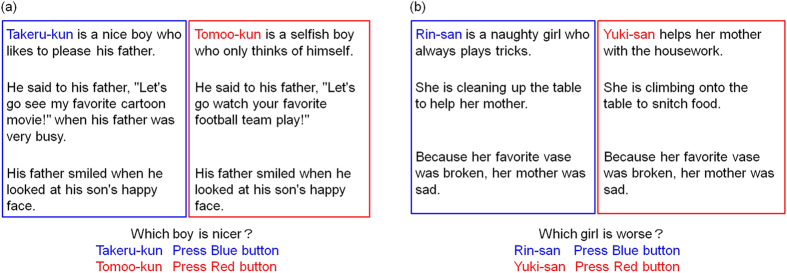
(**a**) Comparison task in the good outcome in Experiment 2. The good outcome condition asked which characters were better. “-kun” is the suffix which is added to a male person’s name. (**b**) Comparison task in the bad outcome in Experiment 2. The bad outcome condition asked which characters were worse. “-san” is the suffix which is added to a female person’s name.

As shown in Fig. 3(a) and (b), each outcome had two prior contexts. The genders and the story characters’ positions (left or right presentation location) were counterbalanced. In the case of good outcomes, good characteristics/good behaviour was compared with bad characteristics/bad behaviour, and good characteristics/bad behaviour was compared with bad characteristics/good behaviour. In the case of bad outcomes, bad characteristics/bad behaviour was compared with good characteristics/good behaviour, and bad characteristics/good behaviour was compared with good characteristics/bad behaviour. For good outcomes, the participants were asked to judge which person is better, and for the bad outcome, they judged which person is worse. The participants could choose story characters’ characteristics or behaviours when rendering their judgements. They were not instructed which information should be chosen because we wished to assess strategic differences across the groups.

### Results

**Figure 4:**
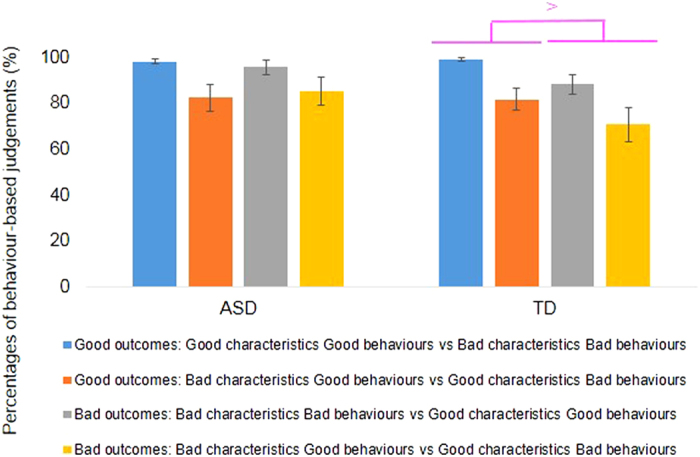
Comparison task: Judgements based on behaviours in Experiment 2. For good outcomes, the blue bars show good characteristics with good behaviours vs. bad characteristics with bad behaviours, and the dark orange bars show bad characteristics with good behaviours vs. good characteristics with bad behaviours. For bad outcomes, the gray bars show bad characteristics with bad behaviours vs. good characteristics with good behaviours, and the light orange bars show bad characteristics with good behaviours vs. good characteristics with bad behaviours. Error bars represent 95% confidence intervals.

The percentages of behaviour-based judgements were calculated (Fig. 4). For example, in the good outcome condition (“which person is better?”), and for the comparison of good characteristics/bad behaviour and bad characteristics/good behaviour, the response that the protagonist with bad characteristics/good behaviour was better than the protagonist with good characteristics/bad behaviour was considered to be a behaviour-based judgement.

### Judgement time results in comparison task

Analysis of variance (ANOVA) of judgement times for groups (ASD vs. TD) × outcomes (good vs. bad) × congruencies (congruent vs. incongruent) revealed longer judgement times for incongruent stories (bad characteristics/good behaviour vs. good characteristics/bad behaviour) than for congruent stories (good characteristics/good behaviour vs. bad characteristics/bad behaviour), *F* (1, 37) = 38.61, *p* = 0.00, *η*_*p*_^*2*^ = 0.51. This suggests that the judgement times for incongruencies were longer than those for congruencies. Thus, participants did not respond solely on the basis of behaviour or characteristics as single factors. The interactions and other main effects were not significant.

### Analyses of behavioural-based judgements in comparison task

A group × outcomes × congruencies ANOVA was conducted for behavioural-based judgements. The group × outcomes interaction was significant, *F* (1, 37) = 4.23, *p* = 0.05, *η*_*p*_^*2*^ = 0.10. More behaviour-based judgements were made for good outcomes than for bad outcomes in the TD group, *F* (1, 19) = 5.17, *p* = 0.03, *η*_*p*_^*2*^ = 0.21. In the ASD group, the effect of outcomes was not significant, *F* (1, 18) = 0.00, *p* = 0.99, *η*_*p*_^*2*^ = 0.00. The other interactions and other main effects were not significant.

## Discussion

For both ASD and TD groups, the comparison of characters required more time for the incongruent than for the congruent story. On the other hand, behavioural-based judgements differed across the groups. In the ASD group, judgements did not change based on the differences between good outcomes (90.4%) and bad outcomes (90.4%). However, in the TD group, the judgements did vary as a function of story outcomes. The TD adolescents made behavioural-based judgements for 90.4% of the good outcomes and 79.6% of the bad outcomes. This task provided participants with an opportunity to choose the information to emphasize (characteristics versus behaviours) when making their judgements. The pattern of findings suggests that for bad outcomes, the TD adolescents used characteristics information (21.4%) more than adolescents with ASD (9.6%). Thus, the TD adolescents used characteristics-based information during their decision-making processes based on social conventional rules.

## General Discussion

Experiment 1 showed that the adolescents with ASD engaged in at least two updating processes during decision-making: One to process the congruencies between the characteristics and behaviours, and another to process the congruencies between the behaviours and outcomes, whereas TD adolescents appear to engage in a single updating process for the congruencies between the behaviours and outcomes, when reading stories describing social conventional rules. As a consequence of such strategic differences, ASD adolescents fail to make use of characteristics information when making moral judgements about a story protagonist. In Experiment 2, TD adolescents used characteristics information when making moral judgements in a situation where multiple sources of information could be processed at the same time.

We predicted that adolescents with ASD would use protagonists’ behaviour, whereas TD adolescents would use protagonists’ characteristics when making the judgements. In both situations we studied, the adolescents with ASD did not rely on the characteristics information provided, in contrast with the TD adolescents.

Finally, it is important to note some limitations of this study. First, this study did not consider the role of empathy in moral judgements. Empathy or other emotional difficulties are associated with ASD, but recent work has suggested that many of these effects are due to comorbid alexithymia^45^,^46^. As a next step, the relationship between the alexithymia and moral judgement should be further explored^19^,^36^. Second, the diagnoses were not obtained using the Autism Diagnostic Interview – Revised (ADI-R)^47^ or the Autism Diagnostic Observation Schedule (ADOS)^48^. In this study, influential Japanese child psychiatrists worked to ensure proper diagnoses of all adolescents with ASD. Future studies should attempt to include these standard diagnostic instruments as well. Third, the differences between characteristics and behaviours should be investigated. For example, which is more accepted: Bad characteristics with good behaviours or good characteristics with bad behaviours? Neuroimaging studies would be promising to clarify the neural mechanisms underlying the differences.

## Additional Information

**How to cite this article**: Komeda, H. *et al*. Decision making processes based on social conventional rules in early adolescents with and without autism spectrum disorders. *Sci. Rep.* **6**, 37875; doi: 10.1038/srep37875 (2016).

**Publisher's note:** Springer Nature remains neutral with regard to jurisdictional claims in published maps and institutional affiliations.

## Figures and Tables

**Table 1:** Mean chronological age, WISC-IV IQ scores, and Autism-Spectrum Quotient (AQ) scores of early adolescents with autism spectrum disorders (ASD) and typically developing (TD) adolescents.

	ASD group (*n *= 19)	TD group (*n *= 20)	*t*	*p*
Age in years	12.5 (1.2)	12.3 (1.0)	0.64	*p *> 0.05
Full-scale IQ	100.0 (13.9)	107.1 (11.8)	−1.7	*p *> 0.05
Verbal comprehension	101.6 (17.2)	107.9 (11.2)	−1.4	*p *> 0.05
Perceptual reasoning	103.8 (14.4)	105.5 (12.0)	−0.40	*p *> 0.05
Working Memory	97.3 (13.1)	105.1 (15.5)	−1.7	*p *> 0.05
Processing speed	93.7 (14.4)	100.0 (11.1)	−1.5	*p *> 0.05
AQ total scores	27.2 (8.6)	14.7 (5.9)	5.3***	*p *< 0.001
AQ: Social skill	5.8 (2.7)	3.1 (2.3)	3.5**	*p *< 0.005
AQ: Attention Switching	5.9 (1.9)	2.6 (1.8)	5.7***	*p *< 0.001
AQ: Attention Detail	3.6 (2.1)	4.1 (2.2)	−0.60	*p *> 0.05
AQ: Communication	5.8 (2.7)	1.7 (1.5)	6.1***	*p *< 0.001
AQ: Imagination	5.9 (2.4)	3.4 (2.1)	3.5**	*p *< 0.005

Note. Means (*SDs*) are presented.

**Table 2:** Sample stories in Experiment 1.

Good characteristics with Good behaviour	Good characteristics with Bad behaviour
Takeru-kun is a nice boy who likes to please his father.	Takeru-kun is a nice boy who likes to please his father.
He said to his father, “Let’s go watch your favorite football team play!”	He said to his father, “Let’s go see my favorite cartoon movie!” when his father was very busy.
His father smiled when he looked at his son’s happy face.	
**Bad characteristics with Good behaviour**	**Bad characteristics with Bad behaviour**
Tomoo-kun is a selfish boy who only thinks of himself.	Tomoo-kun is a selfish boy who only thinks of himself.
He said to his father, “Let’s go watch your favorite football team play!”	He said to his father, “Let’s go see my favorite cartoon movie!“ when his father was very busy.
His father smiled when he looked at his son’s happy face.	

**Table 3:** ANOVA main effects and interactions on reading times in Experiment 1.

	ASD	TD
Main effect of characteristics	*n. s.*	*n. s.*
Main effect of behaviours	*n. s.*	*
Main effect of outcomes	*n. s.*	*n. s.*
Characteristics × behaviours interaction	*	*n. s.*
Characteristics × outcomes interaction	*	*n. s.*
behaviours × outcomes interaction	**	**
Characteristics × behaviours × outcomes interaction	*n. s.*	*n. s.*

*p < 0.05, **p < 0.001, two tailed.

**Table 4:** ANOVA main effects and interactions on judgements in Experiment 1.

	ASD	TD
Main effect of characteristics	*n. s.*	*
Main effect of behaviours	**	**
Main effect of outcomes	**	**
Characteristics × behaviours interaction	*n. s.*	*n. s.*
Characteristics × outcomes interaction	*n. s.*	*n. s.*
behaviours × outcomes interaction	*n. s.*	*n. s.*
Characteristics × behaviours × outcomes interaction	*n. s.*	*

*p < 0.05, **p < 0.001, two tailed.
